# Supplementary material for: LINC00969 inhibits proliferation with metastasis of breast cancer by regulating phosphorylation of PI3K/AKT and ILP2 expression through HOXD8
Source: PeerJ. 2023 Dec 18;11:e16679. doi: 10.7717/peerj.16679 (PMC10734406; doi:10.7717/peerj.16679)
Supplement: Figure S1 — (A) The microscope images showed a obviously decrease in the number of cells in the ov-LINC00969 group. (B) Cell apoptosis of MCF-7 cells transfected with ov-LINC00969 or ov-NC plasmids was detected by flow cytometer assay. (C) Cell cycle of MCF-7 cells transfected with ov-LINC00969 or ov-NC plasmids was detected by flow cytometer assay. (magnification, 100×). (***P < 0.001) [file peerj-11-16679-s001.docx]

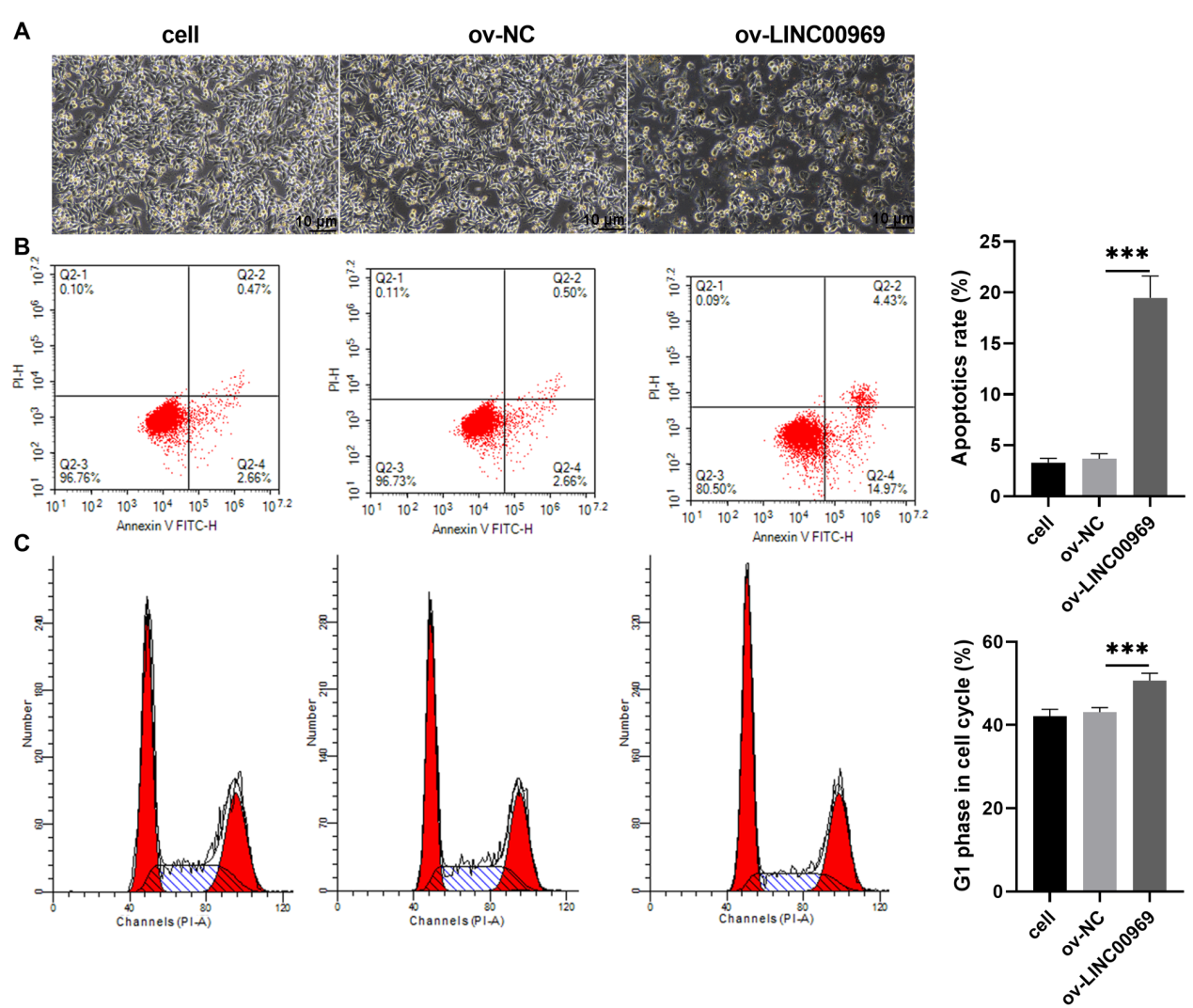


Figure S1 LINC00969 overexpression inhibited the apoptosis and arrested the cell cycle of BC cells. (A) The microscope images showed a obviously decrease in the number of cells in the ov-LINC00969 group. (B) Cell apoptosis of MCF-7 cells transfected with ov-LINC00969 or ov-NC plasmids was detected by flow cytometer assay. (C) Cell cycle of MCF-7 cells transfected with ov-LINC00969 or ov-NC plasmids was detected by flow cytometer assay. (magnification, 100×). (****P* < 0.001).
