# Supplementary material for: LINC00969 inhibits proliferation with metastasis of breast cancer by regulating phosphorylation of PI3K/AKT and ILP2 expression through HOXD8
Source: PeerJ. 2023 Dec 18;11:e16679. doi: 10.7717/peerj.16679 (PMC10734406; doi:10.7717/peerj.16679)
Supplement: Supplemental Information 2 [file peerj-11-16679-s002.pdf]

ITEM TO CHECK	IMPORTANCE	CHECKLIST
**EXPRIMNETAL DESIGN**		
Definition of experiment and control group	E	Cell culture and transfection section, line87-89
Number within each group	E	Cell growth with proliferation assay section, line 131
Assay carried out by core lab or investigator's lab?	D	Western blotting section, line 146
Acknowledgement of author's contributions	D	None
**SAMPLE**		
Description	E	
Volume /mass of sammples processed	D	Cell culture and transfection section, line 89
Microdissection or macrodissection	E	Transwell assay of cell migration and invasion section, line 146
Processing procedure	E	
If frozen-how and how quickly?	E	Quantitative reverse transcription-polymerase chain reaction (qRT-PCR) section, line 99
If fixed-with what how quickly?	E	Quantitative reverse transcription-polymerase chain reaction (qRT-PCR) section, line 101
Sample storage conditions and durarion especially for ffpe sample	E	Quantitative reverse transcription-polymerase chain reaction (qRT-PCR) section, line 101
**NUCLEIC ACID EXTRACTION**		
Procedure and/or details of any modifications	E	Quantitative reverse transcription-polymerase chain reaction (qRT-PCR) section, line 98-104
Name of kit and details of any modifications	E	Quantitative reverse transcription-polymerase chain reaction (qRT-PCR) section, line 99-100
Source of additional reagents used	D	Quantitative reverse transcription-polymerase chain reaction (qRT-PCR) section, line 99
Details of DNase or RNase treatment	E	Quantitative reverse transcription-polymerase chain reaction (qRT-PCR) section, line 99
Contamination assessment (DNA or RNA)	E	Quantitative reverse transcription-polymerase chain reaction (qRT-PCR) section, line 102
Nucleic acid quantification	E	Quantitative reverse transcription-polymerase chain reaction (qRT-PCR) section, line 104
Instrument and method	E	Quantitative reverse transcription-polymerase chain reaction (qRT-PCR) section, line 104
Purity(A260/A280)	D	Quantitative reverse transcription-polymerase chain reaction (qRT-PCR) section, line 100
Yield	D	Quantitative reverse transcription-polymerase chain reaction (qRT-PCR) section, line 101
RNA integrity method/instrument	E	Quantitative reverse transcription-polymerase chain reaction (qRT-PCR) section, line 102
RIN/RQI or Cq of 3’and 5'transcripts	E	Quantitative reverse transcription-polymerase chain reaction (qRT-PCR) section, line 103
Electrophoresis traces	D	Quantitative reverse transcription-polymerase chain reaction (qRT-PCR) section, line 102

Inhibition testing (Cq dilutions, spike or other)	E	Quantitative reverse transcription-polymerase chain reaction (qRT-PCR) section, line 100
**REVERSE TRANSCRIPTION**		
Complete reaction conditions	E	Quantitative reverse transcription-polymerase chain reaction (qRT-PCR) section, line 104-107
Amount of RNA and reaction volume	E	Quantitative reverse transcription-polymerase chain reaction (qRT-PCR) section, line 105
Priming oligonucleotide and concentration	E	Quantitative reverse transcription-polymerase chain reaction (qRT-PCR) section, line 125
Reverse transcriptase and concentrarion	E	Quantitative reverse transcription-polymerase chain reaction (qRT-PCR) section, line 106-107
Temperature and time	E	Quantitative reverse transcription-polymerase chain reaction (qRT-PCR) section, line 115-116
Manufacture of reagents and catalpgue numbers	D	
Cqs with and without RT	D	
Storage conditions od cDNA	D	Quantitative reverse transcription-polymerase chain reaction (qRT-PCR) section, line 101
**qPCR TARGET INFORMATION**		
If multiplex efficiency and LOD of each assay	E	Yes
Sequence accession number	E	Gene ID: 440993
Location of amplicion	D	Quantitative reverse transcription-polymerase chain reaction (qRT-PCR) section, line 109-110
Amplicon length	E	Quantitative reverse transcription-polymerase chain reaction (qRT-PCR) section, line 107
In silico specificity screen	E	Quantitative reverse transcription-polymerase chain reaction (qRT-PCR) section, line 111
Pseudogenes,retropsendogenes or other homologs	D	
Sequence alignment	D	
Secondart structure analysis of amplicon	D	
Location of each primier by exon or intron	E	Quantitative reverse transcription-polymerase chain reaction (qRT-PCR) section, line 117-118
What splice variants are targeted	E	Quantitative reverse transcription-polymerase chain reaction (qRT-PCR) section, line 111-112
**qPCR OLIGONUCLEOTIDES**		
Primer sequences	E	Quantitative reverse transcription-polymerase chai n reaction (qRT-PCR) section, line 118-121
Rtprimer DB Identification number	D	
Probe sequence	D	
Location and identity of any modifications	E	
Manofacture of oligonuclortides	D	
Purification method	D	
**qPCR PROTOCOL**		

Complete reaction conditions	E	Quantitative reverse transcription-polymerase chain reaction (qRT-PCR) section, line 112-114
Reaction volume and amount of cDNA/DNA	E	Quantitative reverse transcription-polymerase chain reaction (qRT-PCR) section, line 105-106
Primer,(probe),mg++,and dNTP concentrations	E	Quantitative reverse transcription-polymerase chain reaction (qRT-PCR) section, line 113, 114, 125
Polymerase identity and concentration	E	Quantitative reverse transcription-polymerase chain reaction (qRT-PCR) section, line 113-114
Buffer/kit identity and manufacturer	E	Quantitative reverse transcription-polymerase chain reaction (qRT-PCR) section, line 113-114
Exact chemical constitution of the buffer	D	
Additives (SYBRGREENI,DMSO)	E	None
Manufacture of plates/tubes and catalog number	D	Quantitative reverse transcription-polymerase chain reaction (qRT-PCR) section, line 114-115
Complete thermocycling parameters	E	Quantitative reverse transcription-polymerase chain reaction (qRT-PCR) section, line 115-116
Reaction setup (manual/robotic)	D	Quantitative reverse transcription-polymerase chain reaction (qRT-PCR) section, line 115-116
Manufacture of qpcr instrument	E	Quantitative reverse transcription-polymerase chain reaction (qRT-PCR) section, line 112-113
**qPCR VALIDATION**		
Evidence of optimasation(from gradients)	D	Quantitative reverse transcription-polymerase chain reaction (qRT-PCR) section, line 116
Specificity(gel,sequence,melt,or digest)	E	Quantitative reverse transcription-polymerase chain reaction (qRT-PCR) section, line 101-102
For SYBR green GREEN I,cq of the NTC	E	
Standard curves with slope and y-intercept	E	Quantitative reverse transcription-polymerase chain reaction (qRT-PCR) section, line 121-122
PCR effiency calculated from slope	E	Quantitative reverse transcription-polymerase chain reaction (qRT-PCR) section, line 126
Confidence interval for pcr efficiency or standard error	D	
r2 of standard curve	E	Quantitative reverse transcription-polymerase chain reaction (qRT-PCR) section, line 126
Linear dynamic range	E	
Cq variation at lower limit	E	
Confidence intervals throughout range	D	
Evidence for limit of detection	E	
If multiplex efficiency and LOD of each assay	E	
**DATA ANALYSIS**		
qPCR analysis program (source,version)	E	Quantitative reverse transcription-polymerase chain reaction (qRT-PCR) section, line 112-116
Cq method determination	E	Quantitative reverse transcription-polymerase chain

		reaction (qRT-PCR) section, line 116
Outlier identification and disposition	E	
Result of NTCs	E	
Justification of number and choice of reference genes	E	
Description of normalisation method	E	Quantitative reverse transcription-polymerase chain reaction (qRT-PCR) section, line 112-116
Number and concordance of biological replicates	D	
Number and stage (RT or qpcr)of technical replicates	E	Quantitative reverse transcription-polymerase chain reaction (qRT-PCR) section, line 127
Repeatability (intra assay variation,)	E	
Repeatability (intra assay variation,%CV)	D	
Power analysis	D	
Statistical methods for result significance	E	Statistical analysis section, line 166.
Software(source,version)	E	Statistical analysis section, line 163-164.
Cq or raw data submission using RDML	D	
