# Supplementary figures and images for: LINC00969 inhibits proliferation with metastasis of breast cancer by regulating phosphorylation of PI3K/AKT and ILP2 expression through HOXD8
Source: PeerJ. 2023 Dec 18;11:e16679. doi: 10.7717/peerj.16679 (PMC10734406; doi:10.7717/peerj.16679)

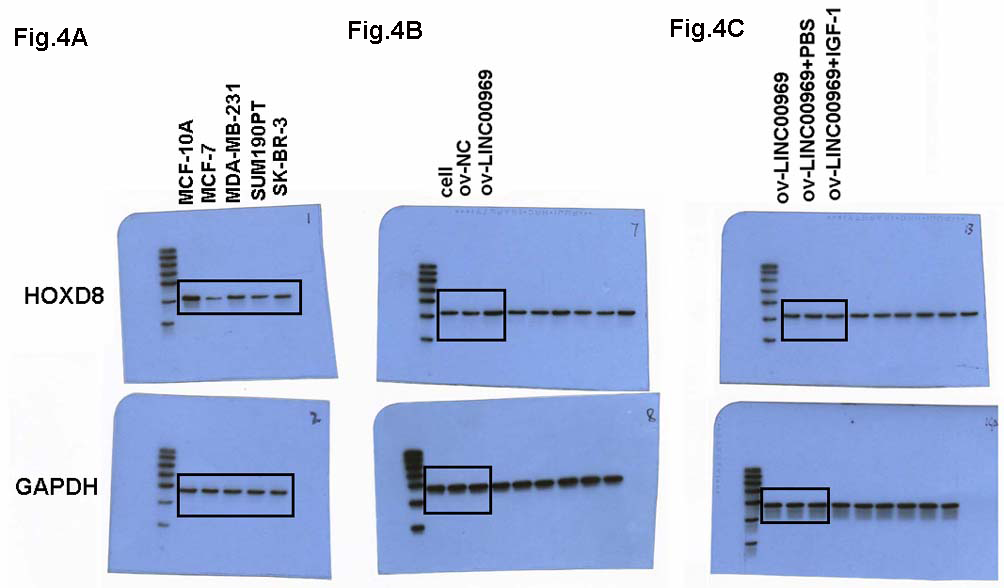

Supplement: Supplemental Information 3 [file peerj-11-16679-s003.zip › Figure 4ABC.png]

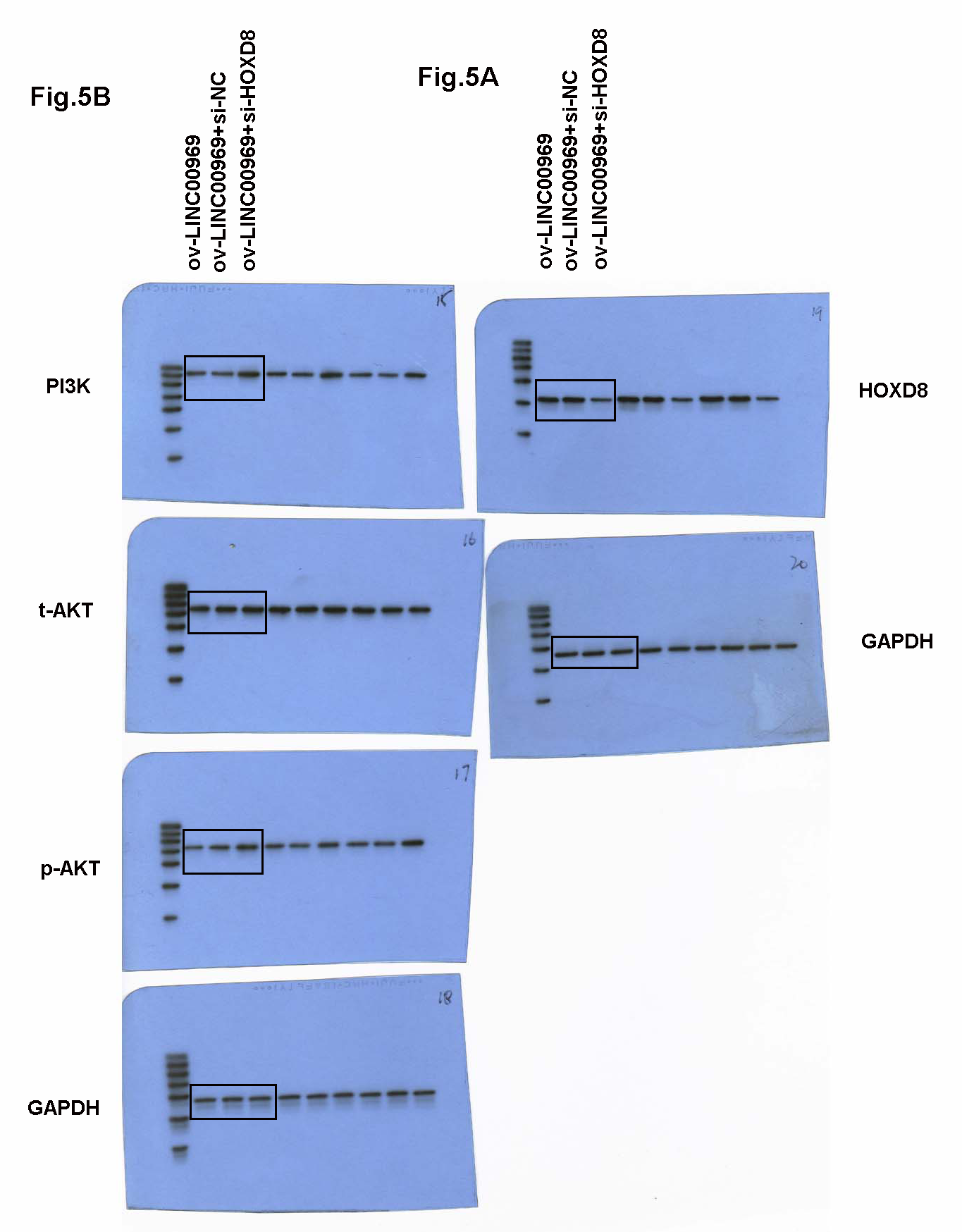

Supplement: Supplemental Information 3 [file peerj-11-16679-s003.zip › Figure 5AB.png]

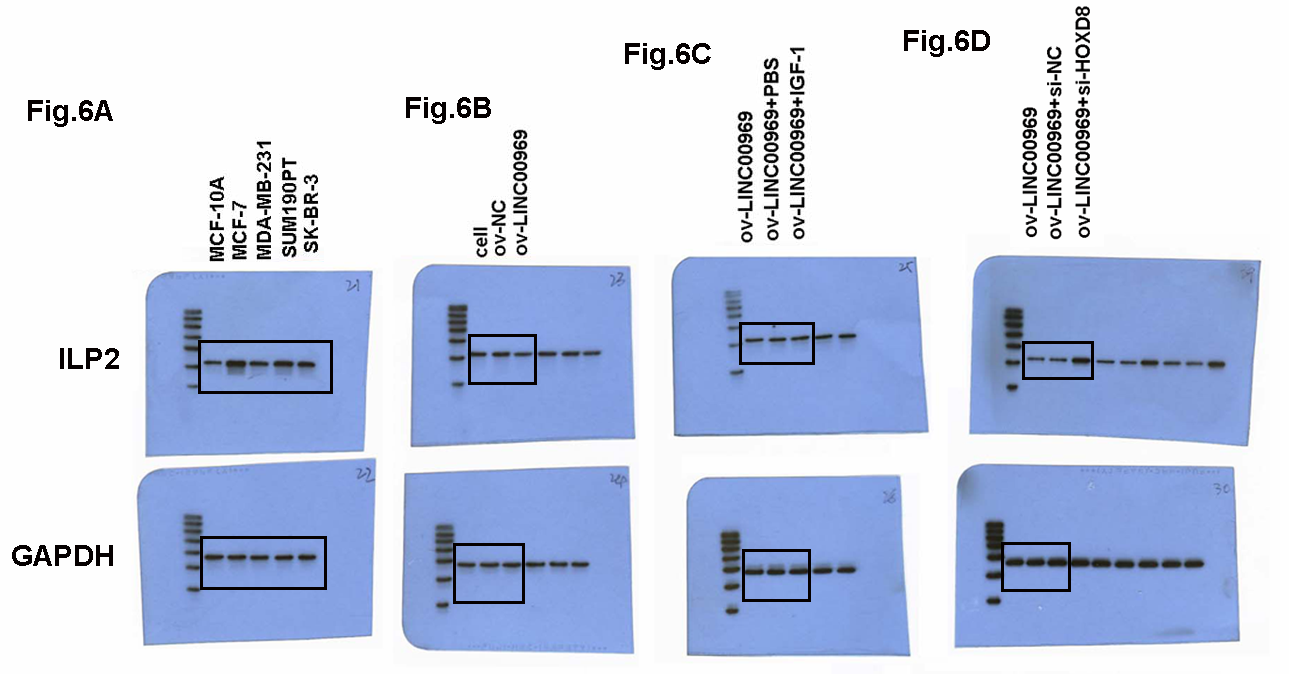

Supplement: Supplemental Information 3 [file peerj-11-16679-s003.zip › Figure 6.png]

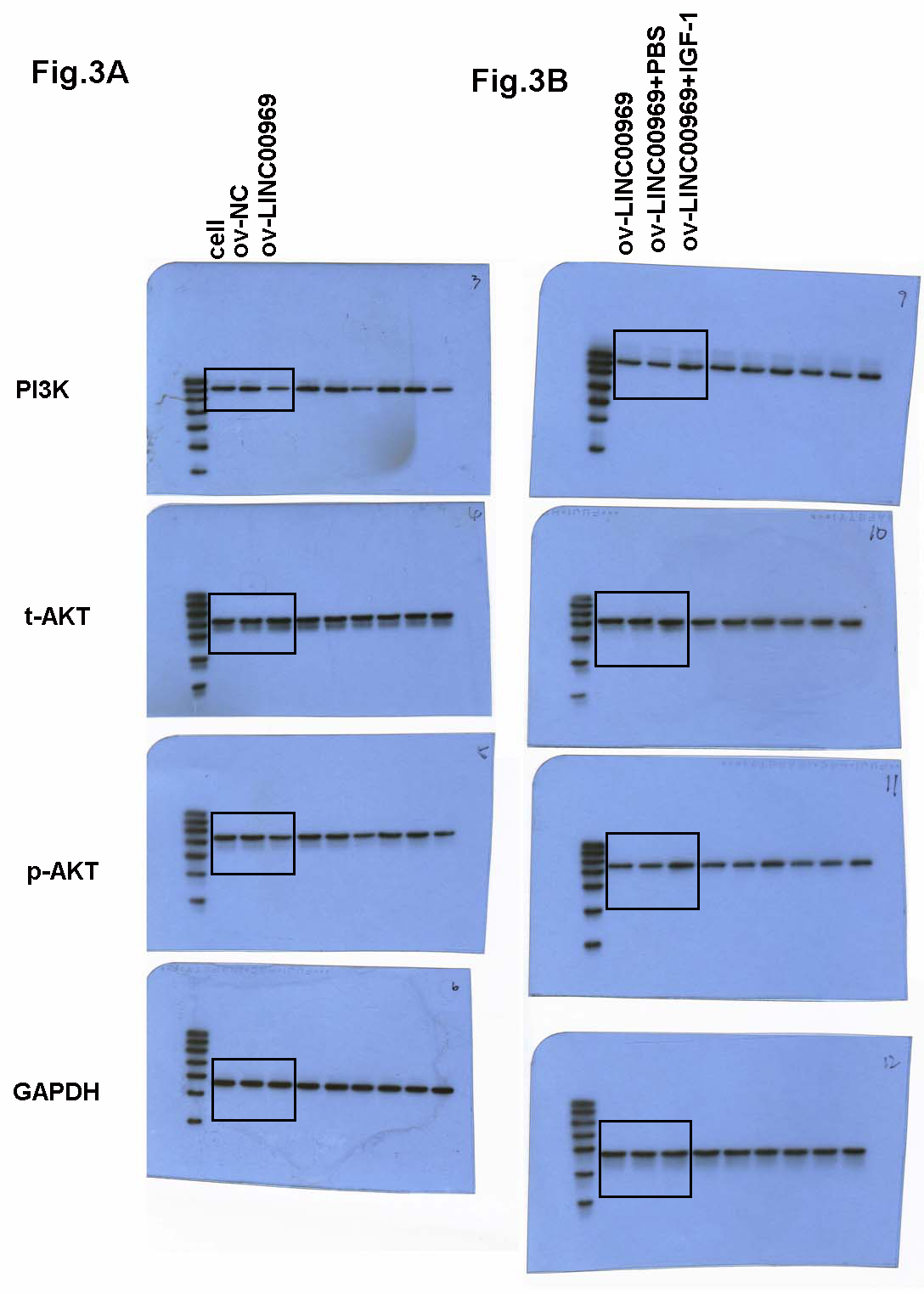

Supplement: Supplemental Information 3 [file peerj-11-16679-s003.zip › Figure 3AB.png]
